# Combination of palbociclib and radiotherapy for glioblastoma

**DOI:** 10.1038/cddiscovery.2017.33

**Published:** 2017-07-03

**Authors:** Shane Whittaker, Daniel Madani, Swapna Joshi, Sylvia A Chung, Terrance Johns, Bryan Day, Mustafa Khasraw, Kerrie L McDonald

**Affiliations:** 1 Cure Brain Cancer Foundation Biomarkers and Translational Research Group, Prince of Wales Clinical School, University of New South Wales Sydney, Sydney, NSW, Australia; 2 Oncogenic Signalling Laboratory, Hudson Institute of Medical Research, Centre for Cancer Research, Melbourne, VIC, Australia; 3 Translational Brain Cancer Research Laboratory, Queensland Institute of Medical Research (QIMR) Berghofer MRI, Brisbane, QLD, Australia; 4 NHMRC Clinical Trials Centre, Chris O’Brien LifeHouse, University of Sydney, Sydney, NSW, Australia

## Abstract

The cyclin-dependent kinase inhibitor, palbociclib has shown compelling efficacy in breast cancer patients. Several pre-clinical studies of glioblastoma (GBM) have also shown palbociclib to be efficacious. In this study, we investigated palbociclib in combination with radiation therapy (RT) for treating GBM. We tested palbociclib (with and without RT) on four patient-derived cell lines (PDCLs; RB1 retained; CDKN2A loss). We investigated the impact of therapy on the cell cycle and apoptosis using flow cytometry, *in vitro*. Balb/c nude mice were intracranially injected with the PDCL, GBM-L1 and treated orally with palbociclib (with and without RT). Overall survival was measured. Palbociclib treatment resulted in a significant increase in the percentage of cells in the G1 cell cycle phase. Apoptotic cell death, measured by Annexin V was induced. Palbociclib combined with RT acted synergistically with the significant impediment of colony formation. The oral treatment of mice with palbociclib did not show any significant survival advantage when compared to control mice, however when combined with RT, a survival advantage of 8 days was observed. Our results support the use of palbociclib as an adjuvant treatment to RT and warrant translation to the clinic.

## Introduction

Glioblastoma (GBM) is a uniformly lethal disease that has had few therapeutic advances over the past century. The cyclin-dependent kinase 4 and 6 (CDK4/6)-retinoblastoma (RB1) signaling pathway is critical to GBM development, presenting itself as a chemotherapeutic target.^1^ The cyclin D-CDK4/6-RB1 axis tightly controls cell cycle progression from the pre-synthetic (G_1_) to DNA synthesis (S) cell cycle phase junctions.^2^ At the nexus of the CDK4/6-RB1 axis is RB1, responsible for regulating cell cycle progression through its inhibitory effect on the E2 transcription growth factor (E2F). E2F propagates key genes involved in cell cycle acceleration, DNA replication and mitotic progression.^3^ Genetic alterations in the CDK4/6 proteins and RB1 are reportedly involved in over 78% of GBM, with prominence in the classical and mesenchymal subtypes of GBM.^4^ Amplification of CDK6 and deletion of the cyclin-dependent kinase inhibitor 2A/B (CDKN2A/B) genes are frequently reported aberrations in primary GBM.^5^

Small molecule CDK inhibitors interact with the catalytic subunit of CDK resulting in the retention of the cyclin proteins in an unphosphorylated state, hence no cyclin–CDK complex form. RB1 phosphorylation is reduced and ultimately transcriptional repression of proliferative genes ensues, resulting in G_1_-phase cell cycle arrest.^6,7^ Palbociclib (PD0332991; Pfizer) is an orally available pyridopyrimidine derivative that selectively inhibits CDK4/6 in RB1 proficient cells. RB1 status is a determinant of tumor therapeutic efficacy for inhibitors that target cyclin-dependent kinases 4 and 6 (CDK4/6). Approximately 11% of GBM show complete loss of RB1 transcript expression^8^ rendering them resistant to CDK4/6 inhibition.

Palbociclib has previously been shown to inhibit the growth of intracranial GBM xenografts.^9–11^ Palbociclib has been found to sufficiently cross the blood brain barrier^9^ at effective concentrations to achieve an anti-proliferative effect. Synergy between palbociclib and RT has also been reported.^9,11^ Herein, we evaluated the efficacy of palbociclib on a panel of four patient-derived cell lines (PDCLs), all with proficient RB1 status and deletion of CDKN2A. We measured the rate of apoptosis within the treated PDCLs and the ability of palbociclib to cause G1-phase cell cycle arrest. We treated intracranially injected Balb/c nude mice with concurrent palbociclib and radiation, confirming that this is an effective combination for development into a clinical trial.

## Results

### Effect of palbociclib as a monotherapy on PDCLs

**Figure 1:**
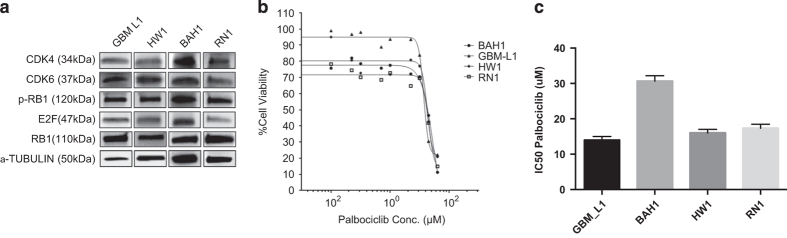
Treatment of patient-derived cell lines (PDCLs) with palbociclib. (**a**) Expression of retinoblastoma protein (Rb1) pathway proteins in PDCLs without treatment; (**b**) Dose-response curves of the PDCLs treated with increasing concentrations of palbociclib; (**c**) Median IC_50_ doses of palbociblib for each PDCL. All experiments were repeated three times. Error bars represent the standard deviation of the mean.

To assess for stemness, all untreated PDCLs were stained with glial fibrillary acid protein (GFAP). Cells were forced to differentiate upon removal of the growth factors; epidermal growth factor (EGF) and fibroblast growth factor (FGF). Supplementary Figure 1 demonstrates the increased expression of GFAP in the differentiated cell line, RN1. Protein was extracted from untreated PDCLs, GBM-L1, HW1, BAH1 and RN1 to establish RB1 proficiency. As can be seen in Figure 1a, all cell lines expressed RB1 and its phosphorylated form. We also determined the protein expression levels of CDK4, CDK6 and E2F. In all 4 cell lines, expression levels of CDK4, CDK6 and E2F were detectable. The expression of CDKN2A was absent in all 4 cell lines (data not shown). We tested a concentration range of palbociclib across the 4 cell lines (Figures 1b and c). The IC50 values ranged from 11 *μ*M for GBM-L1 to 31 *μ*M for BAH1. These IC50 values are appreciably high compared to similar studies in breast cancer.^12^

### Palbociclib induces cell cycle arrest and apoptosis

**Figure 2:**
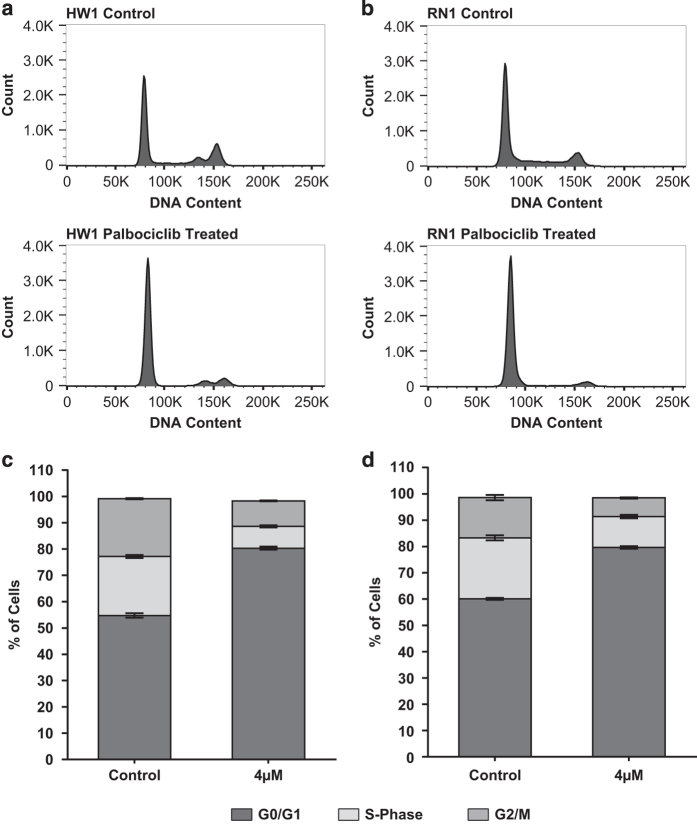
Distribution of cell cycle phase in HW1 and RN1 cell lines treated with palbociclib. (**a**, **b**) DNA histograms generated by flow cytometry showing the distribution of cells in various stages of the cell cycle for cell lines HW1 and RN1. Cells were treated with either DMSO vehicle (control) or 4 *μ*M palbociclib (treated) for 48 h. Histograms are representative of *n*=3 experiments. (**c**, **d**) Graphical representation of DNA histograms for HW1 and RN1 cell lines showing the percentage of cells in the G0/G1, S-Phase and G2/M phases of the cell cycle (*n*=3 experiments).

Palbociclib suppresses DNA replication by preventing cells from entering S-phase. We treated the 4 PDCLs with low dose palbociclib (4 *μ*M) for 24 h. The number of cells in the G0/G1-phase of the cell cycle significantly increased after treatment (Figures 2a–d). The percentage of cells in the G1 cell cycle phase increased from 53 to 81% after treatment of HW1 cells with palbociclib (Figures 2a and c). Similar increases in G1 cell cycle phase were found with the other cell lines (Figures 2b and d showing values for RN1).

**Figure 3:**
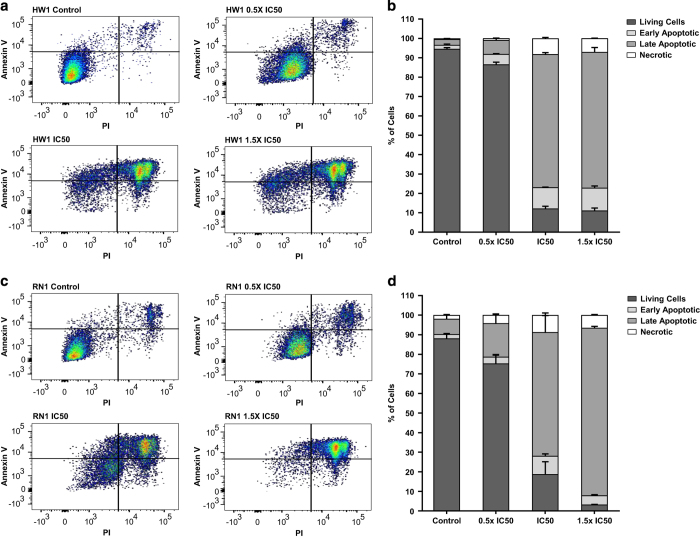
Analysis of apoptosis in HW1 and RN1 cell lines treated with palbociclib. (**a**, **c**) Flow cytometric analysis of apoptosis using annexin V/PI staining of HW1 and RN1 cell lines treated with 0, 0.5, 1 and 1.5× their respective IC50 concentrations of palbociclib for 72 h. Dot-plot images are representative of *n*=3 experiments. (**b**, **d**) Graphical representation of flow cytometric data for HW1 and RN1 cell lines showing the percentage of live, early apoptotic, late apoptotic and necrotic cells (*n*=3 experiments).

Significant increases in apoptosis, measured by Annexin V externalization, were observed when cells were treated with the respective IC50 doses of palbociclib (Figure 3). For HW1 cells, approximately 4% of cells were undergoing apoptosis and this percentage increased to 83% after treatment with the IC50 dose of palbociclib (12 *μ*M; Figures 3a and b). Similar increases in apoptosis were observed with the three cell lines; RN1, BAH1 and GBM-L1. The values for RN1 are shown in Figures 3a and b.

### Palbociclib synergizes with RT to prevent colony formation

**Figure 4:**
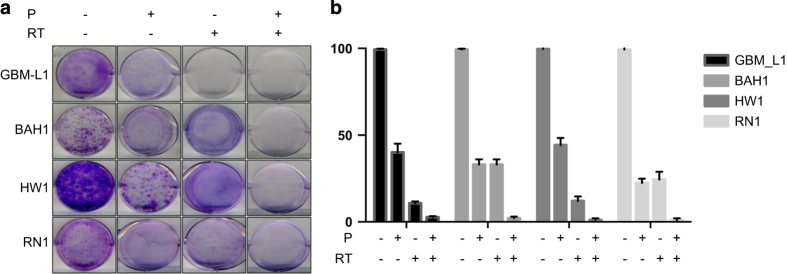
Analysis of colony formation with or without palbociclib treatment of irradiated PDCLs. (**a**) Colony formation assay; (**b**) Colonies were counted from quadruplicate samples for each treatment condition and represented as a percentage of the control (DMSO treated cells). Mean and SD are shown.

**Figure 5:**
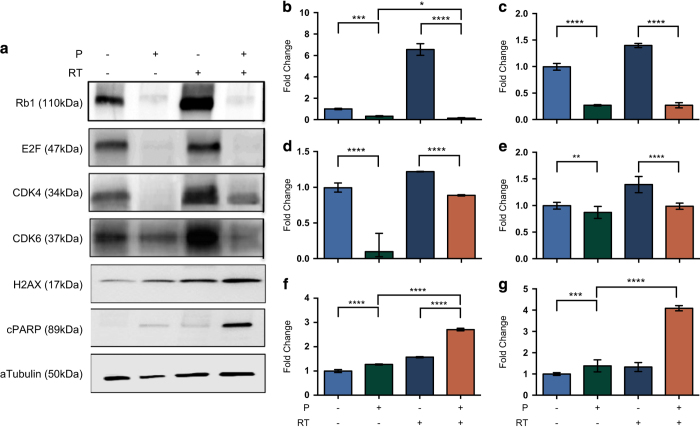
Protein expression in response to treatment with palbociclib with or without RT. (**a**) Representative western blot and protein intensities determined using Image J software (NIH, Bethesda, MD, USA); (**b**) Rb1; (**c**) E2F; (**d**) CDK4; (**e**) CDK6; (**f**) H2AX; (**g**) cPARP. *P<0.05; **P<0.01; ***P<0.005; ****P<0.001.

We combined the IC50 doses of palbociclib with 4 Gy radiation and examined the ability of the cells to form colonies (Figure 4). In all cell lines, palbociclib alone and radiation treatment alone significantly inhibited the formation of colonies. However, when we combined the two treatments, no colonies were detected for any of the cell lines (Figure 4b). We examined the impact of combining palbociclib with radiotherapy on key proteins including phosphorylated RB1, E2F and CDK4 and 6. As can be seen in Figures 5a–e, the addition of radiation treatment to palbociclib did not show an additive effect on the expression of phosphorylated Rb1, E2F and CDK4 and 6. Palbociclib alone was potent enough to suppress the expression of these proteins. However, we did observe increases in *γ*H2AX, a marker of DNA damage and cleaved PARP (cPARP), a measure of apoptosis when palbociclib and radiation treatment were combined (Figures 5a, f and g).

### Palbociclib combined with RT increases survival in an orthotopic GBM model

**Figure 6:**
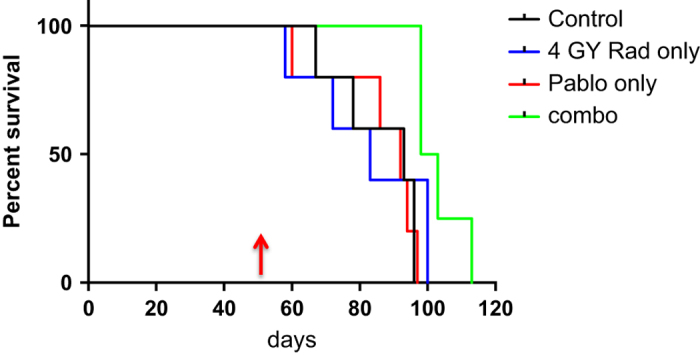
Kaplan–Meier Survival curve for treatments of mice with intracranial RN1 xenografts. Mice were treated at day 55 (indicated by arrow) and received two weeks of palbociclib treatment. Mice were irradiated on days 55 and 56 to receive a total of 4 Gy. LogRank *P*=0.048.

RN1 cells were intracranially injected into balb/c nude mice and allowed to grow for 55 days to form a visible tumor by H&E (mice were humanely euthanized to detect tumor growth). Mice were treated by gavage with palbociclib (75 mg/kg/daily), whole brain radiotherapy (4 Gy) or a combination of both palbociclib and radiotherapy and their survival was compared to control mice (gavaged with saline daily). Treatment was maintained for two weeks. Figure 6 displays the Kaplan–Meier survival curves. No significant differences were observed between the mice treated with palbociclib alone (92 days median survival), radiotherapy alone (83 days median survival) or the control mice (92 days median survival). However, a significant extension in survival times of approximately 8 days was observed in the combination group (palbociclib plus radiotherapy) (100.5 days median survival) (LogRank *P*=0.048).

## Discussion

Amplification of CDK6 and deletion of the cyclin-dependent kinase inhibitor 2A/B (CDKN2A/B) genes are frequently reported aberrations in primary GBM^5^ thus rendering this solid tumor as a potential target for therapeutic intervention with palbociclib. It was recently reported that the addition of RT to palbociclib further sensitized GBM tumors to treatment.^11^ Our pre-clinical studies using PDCLs also confirm this.

Palbociclib treatment effectively prevented cells from entering S-phase of the cell cycle and induced apoptotic cell death. We also demonstrated that palbociclib inhibited colony formation and key components of the cell cycle, namely phosphorylated RB1, E2F and CDK4/6 were directly suppressed. With the addition of RT to palbociclib, increases in the DNA damage marker, γH2AX and the apoptotic marker, cleaved PARP were evident. Critical to the translation to the clinic, it needs to be demonstrated that palbociclib can cross the blood brain barrier. Conflicting evidence is available. While Hashizume and colleagues reported sufficient levels of palbociclib in the brain,^11^ the same group reported earlier results that demonstrated that palbociclib was a substrate for both P-glycoprotein and breast cancer resistance protein^10^ and levels were 115-fold less than the transporter deficient mice when compared with wild-type mice. We did not measure the levels of palbociclib in the brain of orthotopic mice in our current study, however the amount of drug used (75 mg/kg/day) was sufficient to achieve an anti-proliferative effect and a significant extension in survival when combined with RT.

Palbociclib in combination with RT has not been used in the clinic to date for patients diagnosed with GBM. The combination has been trialed in pre-clinical models of medulloblastoma^13^ and brain stem cancer.^14^ The pre-treatment of medulloblastoma cell lines with palbociclib sensitized medulloblastoma cells to ionizing radiation.^13^ Using a genetically engineered brain stem model, investigators found that by priming the cells with radiation (10 Gy) followed by 7 days of palbociclib treatment, survival was increased by 19% in comparison to RT alone.^14^ The order of RT first, followed by palbociclib treatment was found to be highly important in the studies conducted by Hashizume and colleagues.^11^ In the current study, we also treated the patient-derived cells and xenografts mice with RT first, followed by palbociclib treatment.

There is great excitement surrounding the use of palbociclib alone or in combination with other agents in the clinic. The results of the Phase 3 trial, PALOMA-2 (NCT01740427) were recently reported.^15^ Postmenopausal women with ER-positive, HER2-negative breast cancer were treated with palbociclib plus letrozole or placebo plus letrozole. Progression-free survival (PFS) was 24.8 months for patients treated with palbociclib and letrozole compared to 14.5 months for patients treated with the placebo plus letrozole.^15^ A significant caveat of the study was a high proportion of patients treated with palbociclib and letrozole suffered from neutropenia (66.4%). This will greatly impact on future trials for GBM patients.

Moreover, our pre-clinical studies of palbociclib in combination with RT have provided compelling evidence that this combination is efficacious in GBM. Our results support further investigation into the use of CDK inhibitors for GBM that retain RB1 expression and the clinical translation of palbociclib as an adjuvant to RT.

## Materials and methods

### Ethics statement

All orthotopic animal studies were approved by the UNSW Animal Care and Ethics Committee (ACEC).

### Patient-derived cell lines

GBM-L1, HW1, RN1 and BAH1 cell lines were grown in RHB-A medium (Clontech Laboratories, Inc., Mountain View, CA, USA) supplemented with human Epidermal Growth Factor (20 ng/ml; Sigma-Aldrich, St Louis, MO, USA) and human Fibroblast Growth Factor—Basic (20 ng/ml; Sigma-Aldrich), in tissue culture flasks coated with a layer of BD Matrigel Basement Membrane Matrix (1:100 in PBS; BD Biosciences, North Ryde, NSW, Australia). Cells were maintained in a 37 °C, 5% CO_2_ incubator (Thermo Fisher Scientific, North Ryde, NSW, Australia).

### Colony formation assay

Colony formation assays for the PDCLs were performed as previously described.^16^ PDCLs were plated in triplicate in 6-well plates and incubated overnight. The cells were treated with vehicle control (DMSO) or palbociclib in supplemented RBH-A medium. Radiation was delivered using a self-contained X-ray system (X-RAD 320). Plates were incubated for 2 weeks undisturbed. Colonies were gently washed with PBS followed by staining and fixation with crystal violet solution (0.5% in H_2_O:methanol, 1 : 1) for 15 min. Stained colonies consisting of >50 cells were counted and the number was recorded. Plating efficiency was calculated as the number of colonies counted divided by the number of cells seeded and normalized to the average plating efficiency of untreated samples. The average of these values was reported as 'percentage of cells survived compared to the control.'

### Cell proliferation assay

The optimum cell density of each PDCL was established using the MTS, CellTiter 96 Aqueous Assay (Promega, Alexandria, NSW, Australia) and viability was measured 72-h post treatment. PDCLs were treated with increasing concentrations of palbociclib to determine the cytotoxic effects of the chemotherapeutic drugs, and the half-maximal inhibitory concentration (IC_50_).

### Flow cytometry analysis

PDCL’s were seeded into six-well plates at a density of 2×10^5^ cells per well. For cell cycle analysis, cells were cultured in growth media supplemented with 4 *μ*M palbociclib for 48 h. Cells were harvested, washed once in in PBS and then fixed in 70% ethanol for 30 min at 4 ^o^C. Fixed cells were then pelleted, washed twice with PBS, and then resuspended in 400 *μ*l of staining solution containing 50 *μ*g/ml propidium iodide (Sigma-Aldrich) and 100 *μ*g/ml DNase-free RNase (Roche, North Ryde, NSW, Australia). Cells were stained for 30 min in the dark at room temperature. For analysis of apoptosis, cells were cultured in growth media containing either 0x, 0.5x, 1x or 1.5x their respective IC50 values for palbociclib as determined previously. Cells were harvested after 72 h and stained with Annexin V and propidium iodide using an Annexin V-FLUOS Staining Kit (Roche) as per the manufacturer’s protocol. Both the cell cycle and apoptotic assays were performed via flow cytometry using a BD FACSCanto II system (BD Biosciences) and the data obtained analyzed using the FlowJo software (BD Biosciences). For cell cycle analysis, single cells were discriminated from doublets via gating using the FL2W *versus* FL2A for the PI stain. Estimation of distinct cell cycle phases was performed by use of the univariate Watson (pragmatic) model contained within the FlowJo software.

### Western blot

Protein was extracted from untreated PDCLs using cell lysis buffer (10 mM Tris-Cl pH 7.4, 100 mM NaCl, 1 mM EDTA pH 8.0, 1 mM NaF, 20 mM Na_4_P_2_O_7_, 0.1% SDS, 0.5% sodium deoxycholate, 1% Triton X-100, 10% Glycerol, Milli-Q water) and cOmplete, mini, EDTA-free protease inhibitor tablets (Sigma-Aldrich). Western blots were probed with antibodies against CDK4 (Cell Signaling, Danvers, MA, USA; 1 : 2000); CDK6 (Cell Signaling; 1 : 2000); phosphorylated RB1 (Cell Signaling; 1 : 1000); E2F (Cell Signaling; 1 : 1000); RB1 (Abcam, Cambridge, MA, USA; 1 : 1000); CDKN2A/p16 (Abcam; 1 : 1000); gamma histone H2A.X (H2AX; Cell Signaling; 1 : 1000); cleaved PARP (Cell Signaling; 1 : 1000). To control for protein loading, membranes were probed with alpha-tubulin (Abcam; 1 : 10 000).

### *In vivo* experiments

Female athymic nude mice (Balb/c; 8–9 weeks of age) were intracranially injected with 2×10^5^ GBM-L1 PDCLs stereotactically in the right caudate putamen using the coordinates: 1 mm anterior, 1.5 mm lateral and 3.0 mm below the bregma. To monitor tumor growth, animals were humanely euthanized at the following time-points: 40 days, 45 days, 50 days and 60 days. Mouse brains were fixed in formalin and embedded in paraffin. H&E stains of the brains revealed tumor growth by day 50, indicating the time of commencement of treatment. Mice were randomly assigned to 4 groups; (1) untreated control (*n*=9); (2) palbociclib (75 mg/kg per day for 5 days in a two week treatment cycle) (*n*=9); (3) radiation treatment (total of 4 Gy over 2 days) (*n*=9) and (4) palbociclib combined with radiation (*n*=9). Whole brain radiation was delivered using a self-contained X-ray system (X-RAD 320). During RT, mice were placed in a customized lead box to shield the body to allow radiation to be delivered directly to the entire brain. The total radiation dose administered was 4 Gy at a clinically relevant 2 Gy/fraction schedule on 2 consecutive days. One cycle of palbociclib (2 weeks) was administered to the mice, before endpoint was reached.

Mice were euthanized when they exhibited symptoms indicative of significant compromise to neurologic function and/or a greater than 20% body weight loss. Animal survival was defined as the time taken from tumor injection until euthanasia and survival curves were established using the Kaplan–Meier estimator.
