# Supplementary figures and images for: Combination of palbociclib and radiotherapy for glioblastoma
Source: Cell Death Discov. 2017 Jul 3;3:17033–. doi: 10.1038/cddiscovery.2017.33 (PMC5494656; doi:10.1038/cddiscovery.2017.33)

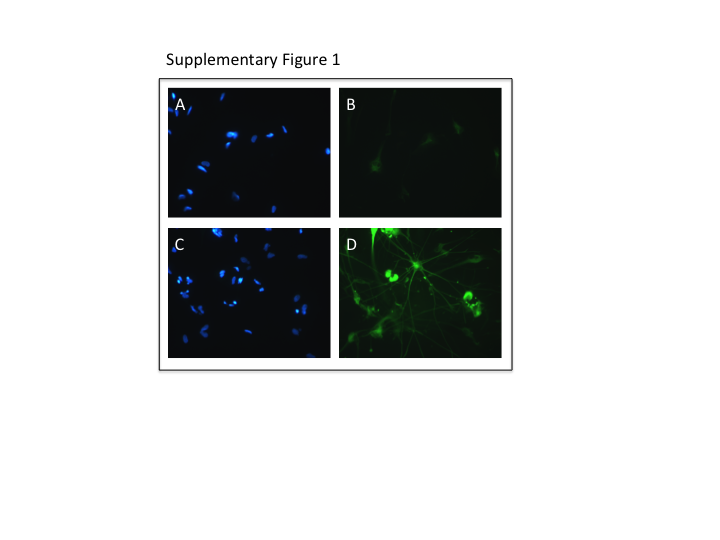

Supplement: Supplementary Figure 1 [file cddiscovery201733-s1.tiff]
